# Autophagy-related genes in mesial temporal lobe epilepsy: an integrated bioinformatics analysis

**DOI:** 10.1186/s42494-024-00160-9

**Published:** 2024-04-25

**Authors:** Man Yang, Yinchao Li, Xianyue Liu, Shangnan Zou, Lei Lei, Qihang Zou, Yaqian Zhang, Yubao Fang, Shuda Chen, Liemin Zhou

**Affiliations:** Department of Neurology, The Seventh Affiliated Hospital, Sun Yat-Sen University, Shenzhen, 518107 Guangdong Province China

**Keywords:** Autophagy, Mesial temporal lobe epilepsy, Bioinformatics analysis, Biomarkers

## Abstract

**Background:**

Autophagy plays essential roles in the development and pathogenesis of mesial temporal lobe epilepsy (mTLE). In this research, we aim to identify and validate the autophagy-related genes associated with mTLE through bioinformatics analysis and experimental validations.

**Methods:**

We obtained the dataset GSE143272 and high-throughput sequencing results of mTLE from public databases. Potential differentially expressed autophagy-related genes related to mTLE were identified using R software. Subsequently, genomes pathway enrichment analysis, protein-protein interactions (PPIs), and the gene ontology (GO) enrichment were performed for the selected autophagy-related genes. The mRNA expression profiles of hub genes were then used to establish a least absolute shrinkage and selection operator (LASSO) model. Finally, seven hub candidate autophagy-related genes were confirmed in hippocampus using the lithium-pilocarpine chronic epilepsy model.

**Results:**

A total of 40 differential expression genes (DEGs) among the core autophagy-related genes were identified. The analysis results of PPI revealed that interactions among these DEGs. KEGG pathway and GO analysis of selected candidate autophagy-related genes indicated that those enriched terms mainly focused on macroautophagy, regulation of autophagy, cellular response to extracellular stimulus and mitochondrion disassembly. The results suggested that *SQSTM1, VEGFA, BNIP* and *WIPI2* were consistent with the bioinformatics analysis. The expression levels of *SQSTM1* and *VEGFA* in epilepsy model samples were significantly higher than those in normal control, while *BNIP* and *WIPI2* expression levels were notably decreased. The final hub gene-based LASSO regression model accurately predicted the occurrence of epilepsy (AUC = 0.88).

**Conclusions:**

Through bioinformatics analysis of public data, we identified 40 candidate autophagy-related genes associated with mTLE. *SQSTM1*, *VEGFA*, *BNIP* and *WIPI2* may play significant roles in autophagy, influencing the onset and development of mTLE by regulating autophagy pathway. These findings deepen our understanding of mTLE, and may serve as sensitive and valuable indicators for the prognosis and diagnosis of this condition.

**Supplementary Information:**

The online version contains supplementary material available at 10.1186/s42494-024-00160-9.

## Background

Epilepsy is a chronic nervous system disorder characterized by recurrent and unprovoked seizures [1]. Temporal lobe epilepsy (TLE) is one of the most common types of epilepsy in adults, often originating in childhood. Mesial temporal lobe epilepsy (mTLE) is usually associated with hippocampal sclerosis (HS), which is the most prevalent neuropathological discovery [2]. Despite being considered as a polygenic and complex disorder, the pathological mechanisms underlying mTLE with HS remain largely unknown. Autophagy, an intracellular catabolic process, plays a crucial role in removing protein aggregates and damaged intracellular organelles by transporting them to lysosomes [3], thereby maintaining cell health and cellular homeostasis. However, abnormal autophagy can lead to various neurological disorders, including epilepsy. Notably, studies have suggested a correlation between autophagy and epilepsy [4], with autophagic impairment being implicated in conditions, like focal cortical dysplasia, tuberous sclerosis complex and Lafora disease [5–7]. Experimental models have shown that suppressing autophagy can induce epilepsy, while rescuing autophagy can prevent it [8, 9]. Interestingly, epilepsy itself can lead to dysregulated autophagy. Xiao et al. observed increased apoptosis and decreased mitochondrial autophagy in the hippocampus of epileptic rats induced lithium chloride pilocarpine injection [10]. Moreover, studies have reported several potential mechanisms through which autophagy may influence epileptogenesis, including aberrant substrate accumulation, the mammalian target of rapamycin (mTOR) pathway, and the formation of epileptogenic networks [11, 12]. However, the role of autophagy in the pathogenesis of epilepsy is still unclear, highlighting the need for further investigation. Therefore, this study aims to identify autophagic candidate genes that may play an important role in the pathogenesis of mTLE, and potentially serving as sensitive indicators for mTLE.

## Methods

### Genes associated with autophagy and microarray data

**Fig. 1:**
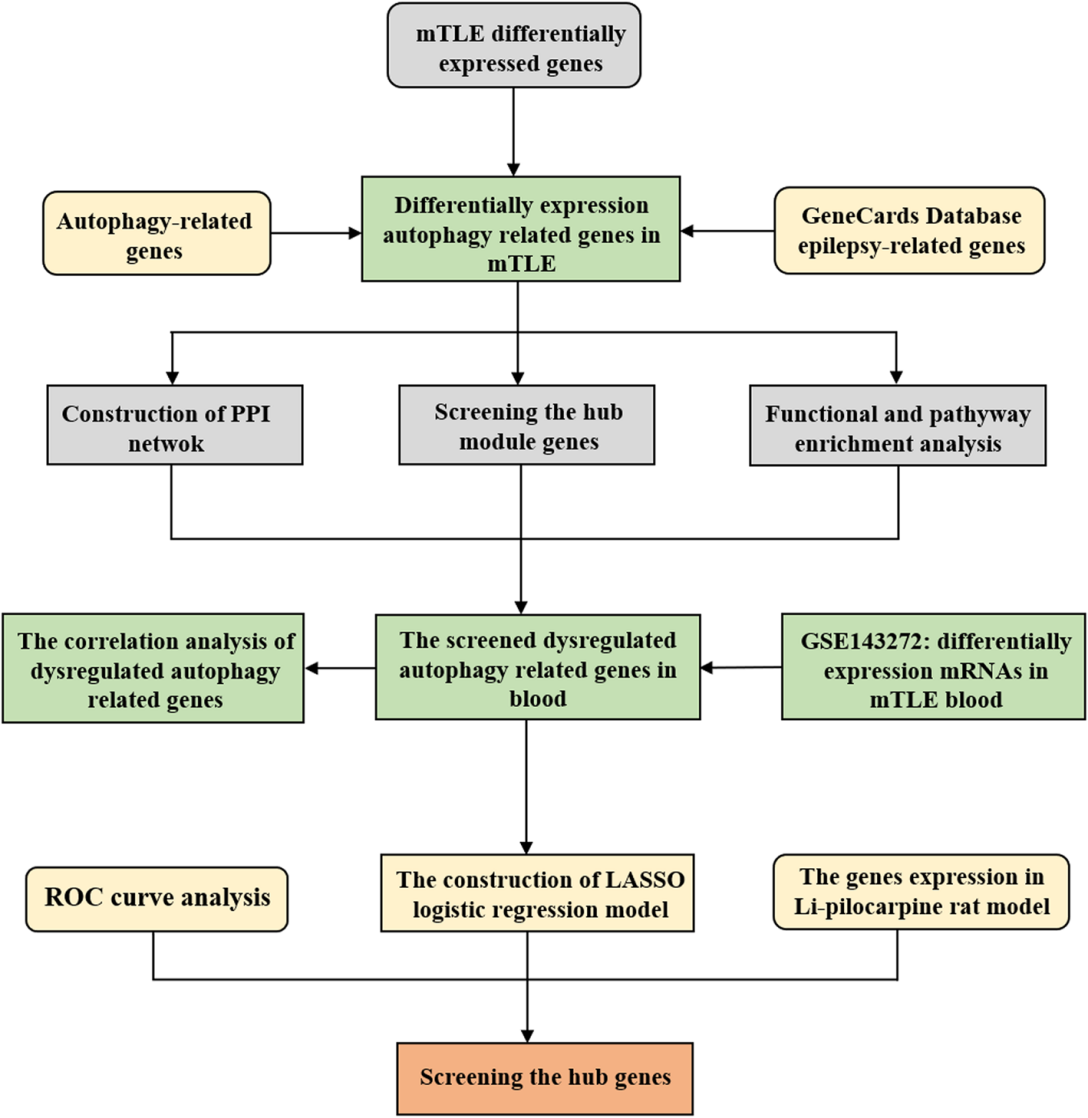
Workflow analysis Abbreviations: LASSO: Least absolute shrinkage and selection operator; mTLE: Mesial temporal lobe epilepsy; PPI: Protein-protein interaction; ROC: Receiver operating characteristic

The research workflow is illustrated in Fig. 1. Candidate autophagy-related genes were identified by searching the Human Autophagy Database. Subsequently, we utilized GeneCards: the Human Gene Database to extract epilepsy medical subject headings (MeSH) terms and other epilepsy-related textual terms. These results were then combined with the previous public high-throughput sequencing data of mTLE to track the expression profiles of mTLE-related autophagy genes [13]. The dataset comprised temporal cortical samples from 75 neurologically healthy individuals and 85 patients with confirmed neuropathological mTLE + HS. Exon and gene-level transcriptome analysis were performed, residual methods were applied to alter the data, and the false detection rate (FDR) threshold was set at 5%.

### Functional enrichment analysis of autophagy-related genes

The candidate autophagy-related genes in mTLE underwent KEGG pathway enrichment and GO functional enrichment analysis using R software. The GO analysis included cellular component (CC), biological process (BP), and molecular function (MF).

### PPI construction and identification of hub module

PPI networks were constructed using the STRING database. Subsequently, the cytoHubba plugin of the Cytoscape software was employed to identify the hub module. The cytoHubba plugin ranks nodes according to their properties in the network, facilitating the identification of key targets and sub-networks within complex networks.

### Screening of differential blood and brain autophagy-related mRNA expression profiles in mTLE

Peripheral blood mRNA expression profiles were acquired from the public database GEO (GSE143272). Total RNA samples were initially collected from peripheral blood samples of 34 drug-naive epilepsy patients and 57 patients exhibiting differential response to anti-seizure medication monotherapy, as well as 50 healthy individuals as controls. Subsequently, the peripheral blood expression profiles of patients with epilepsy receiving or not receiving anti-seizure medication monotherapy and their responses to the treatment were acquired. By comparing the gene expression profiles of drug-naive epilepsy patients with those of healthy controls in the GSE143272 database, mRNA expression biomarkers associated with epilepsy and the anti-seizure medication response were identified. These expression profiles exhibited significant differences and potential differential expression genes (DEGs) among the above three groups of microarray data and were used for further analysis.

### Correlation analysis of the candidate DEGs associated with autophagy

Spearman correlation analysis was conducted using the “corrplot” package in R software to determine the correlations among the identified DEGs associated with autophagy.

### LASSO-Cox regression model analysis

The mRNA expression profiles of the hub genes were extracted to construct a LASSO model. Using the R software package “glmnet”, we integrated the time of onset, the presence of epilepsy, and gene expression data, applying the LASSO-Cox method for regression analysis. A 5-fold cross-validation was implemented in the LASSO-Cox regression model to identify the optimal values.

### Establishment of pilocarpine-induced chronic seizures model

A total of 20 Sprague-dawley (SD) rats, aged 8–9 weeks, male, weighing 160–200 g, were obtained from Beijing Vitleyhua Experimental Animal Co., Ltd. All rats were provided with ad libitum access to food and water. Before the experiment, the rats were adapted to the environment for at least 3 days. All procedures are in line with the “Regulations on Experimental Animal Management”. On the first day of the experiment, the rats were injected with lithium chloride. Then after 24 h, they received an injection of methylbromine (1 mg/kg, Macklin S835305), followed by an abdominal injection of pilocarpine (initial dose of 30 mg/kg, TargetMol T0804) for 30 min. Additional doses of pilocarpine were injected every 30 min at 10 mg/kg increments until the rats reached IV (bilateral forelimb clonus with rearing and falling) or status epileptics (SE) – without obvious intervals, with the maximum dose of 60 mg/ kg. Two hours following the onset of, epileptic seizures, diazepam was administered to terminate the seizures. The symptoms of seizures in rats were graded according to the Racine grading criteria. Subsequently, all rats exhibiting spontaneous recurrent seizures were designated as chronic epilepsy animals and were randomly selected for EEG recording.

### RNA isolation and quantitative reverse-transcription polymerase chain reaction (RT-qPCR)

Total RNA was extracted using the RNA extraction kit (DP501, TIANGEN, China) following the manufacturer’s protocol for RNA RT-qPCR. RNA quantity was determined using Nanodrop (Thermo-Fisher). First-strand cDNA synthesis was carried out with the cDNA synthesis kit (KR118, TIANGEN, China) according to the manufacturer’s instructions. The qPCRs were conducted in a Quant-studio 6 flex Real-Time PCR system (Applied Biosystems) with SYBR Green kit (FP209, TIANGEN, China). The primers used were: 5’− GCTGGTTCGTGGTGGACTTCATC − 3’ and 5’− TGCTCTGGCGGTCTTGTAAACTTC − 3’ (Sangon Biotech, China). β-actin primers were used as a reference (B661202 Sangon Biotech). The relative expression levels were calculated using the 2^−ΔΔCt^ method.

### Statistical analysis

The *t*-test was performed using GraphPad Prism 9 software. For differential gene expression analysis, a criteria of |log2FC| > 0.5, and a *P*-value less than 0.05 was considered to be statistically significant.

## Results

### Genes associated with autophagy in mTLE

**Fig. 2:**
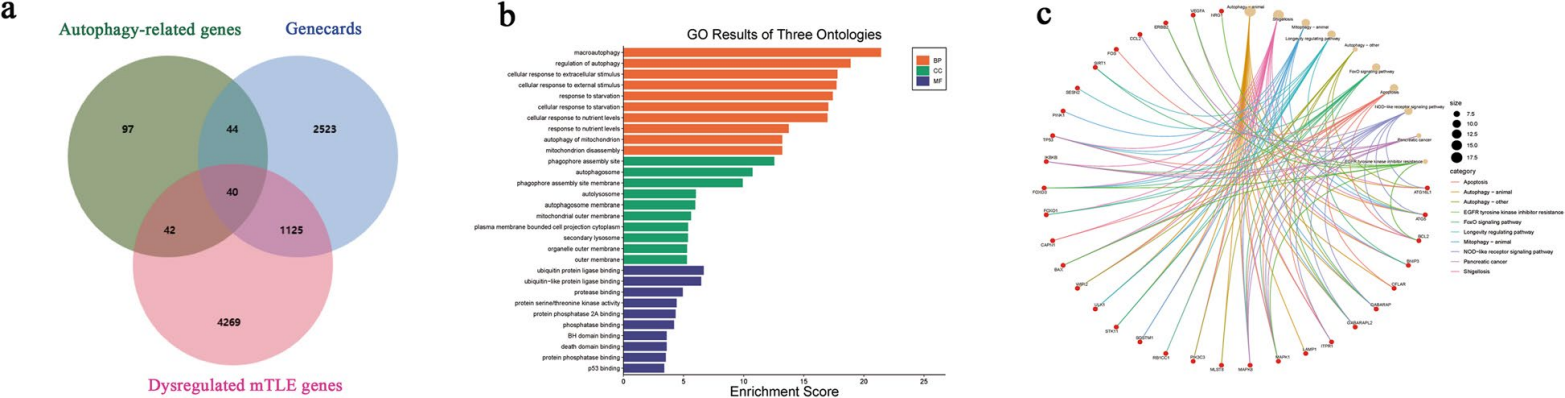
Identification of autophagy-related genes and functional enrichment analysis. **a** Screening autophagy-related DEGs in mTLE. **b** GO enrichment analysis of genes associated with autophagy in mTLE. **c** Chord diagram illustrating top 10 KEGG enrichment analysis results

A total of 233 autophagy-related genes were initially identified from the HADb. Subsequently, 40 autophagy-related genes were selected based on differential expression mTLE, comprising 23 upregulated genes and 17 downregulated genes. The summary of these findings is presented in Fig. 2a, with detailed information available in Supplement Table 1.

### Functional and pathway enrichment analysis

The 40 differentially expressed autophagy-related genes associated with mTLE were included in KEGG pathway and GO term analysis. The top enriched BP included cellular response to extracellular stimulus, regulation of autophagy, macroautophagy, and mitochondrion disassembly. GO terms related to CC focused on phagophore assembly site, autophagosome, and plasma membrane bounded cell projection cytoplasm. GO terms related to MF included protein serine/threonine kinase activity, ubiquitin protein ligase binding, and protease binding. Moreover, KEGG pathway enrichment analysis identified FoxO signaling pathway, autophagy, apoptosis and mTOR signaling pathway (Fig. 2b and c).

### Identification of the hub module of mTLE autophagy-related genes

**Fig. 3:**
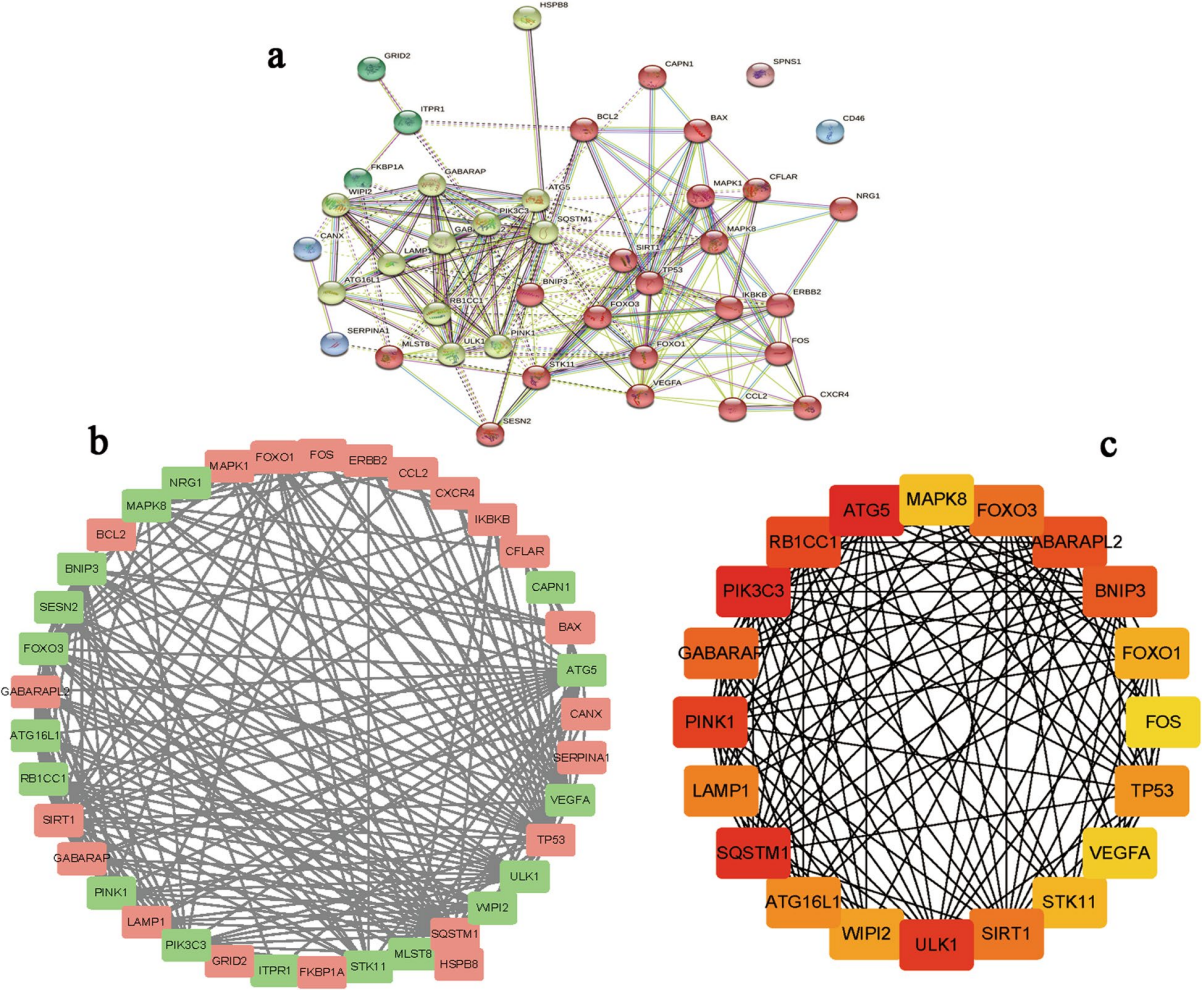
PPI network of autophagy related candidate genes and central module in mTLE. **a** Autophagy-related genes associated with mTLE were categorized into three major modules. **b** The PPI network of mTLE autophagy-related genes depicted red dots representing up-regulated genes, green dots representing down-regulated genes, and black lines indicating interactions between the encoded proteins. **c** The PPI network of hub module genes

With the application of STRING database, PPI network of autophagy related genes was constructed. Next, the MCODE plugin was applied for module analysis. The PPI network was primarily divided into three main modules (Fig. 3a and b). Among these modules, a hub module consisting of 20 genes with high connectivity throughout the PPI network was identified and may play a crucial role in physiological processes (Fig. 3c; Table 1).

**Table 1:** The hub module genes in mTLE

Genes	* P*-value	LogFC	Expression
*BNIP3*	0.00009	-0.26286	Down
*SQSTM1*	0.00062	0.13393	Up
*WIPI2*	0.00078	0.19649	Up
*FOXO3*	0.00185	0.19416	Up
*PINK1*	0.00191	0.22349	Up
*VEGFA*	0.00996	0.16619	Up
*ATG5*	0.01510	-0.11207	Down
*GABARAPL2*	0.03950	0.10655	Up
*ATG16L1*	0.06370	-0.07946	Down
*MAPK8*	0.06600	-0.11502	Down
*LAMP1*	0.14400	0.05001	Up
*FOS*	0.16300	0.18538	Up
*ULK1*	0.24800	0.07328	Up
*STK11*	0.39800	0.04798	Up
*PIK3C3*	0.50500	0.03688	Up
*GABARAP*	0.54600	-0.04359	Down
*SIRT1*	0.64800	0.02498	Up
*RB1CC1*	0.75000	0.01892	Up
*FOXO1*	0.90000	-0.00659	Down
*TP53*	0.12900	0.17880	Up

### Screening of differential blood and brain autophagy-related gene expression profiles and correlation analysis of the DEGs

**Fig. 4:**
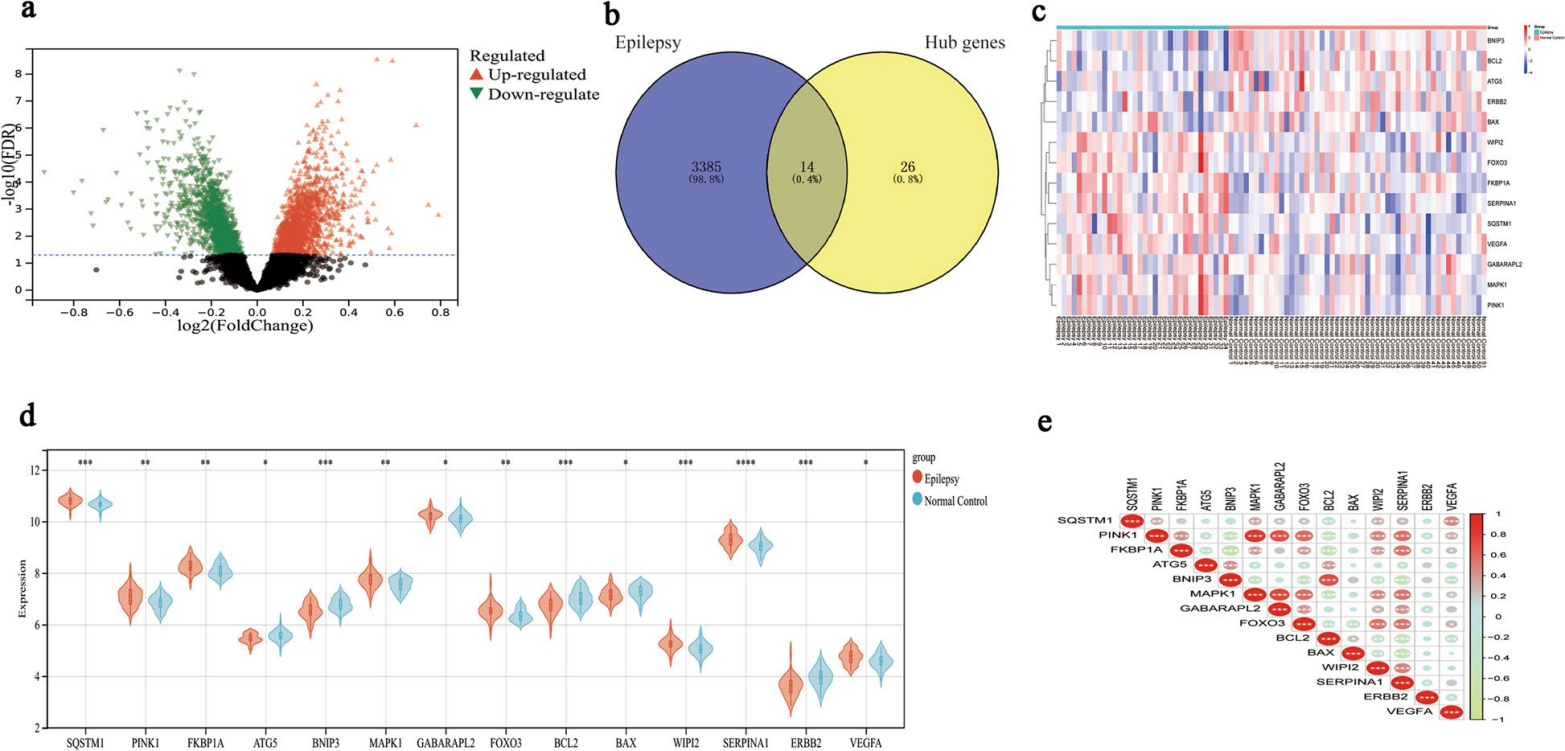
DEGs of autophagy and Spearman correlation analysis of the 14 hub genes. **a** A Volcano plot of the 3399 autophagy-related DEGs in the blood of patients with epilepsy. **b** Venn diagrams of the screened dysregulated genes in epilepsy and the hub genes. The blue part indicates the epilepsy-related genes in blood and brain tissue, while the yellow part indicates the hub genes. **c** A heatmap of the 14 autophagy-related DEGs in mTLE and healthy controls. **d** The violin plots of 14 autophagy-related DEGs in blood of epilepsy patients and controls. **e** Spearman correlation analysis of the 14 autophagy-related DEGs

Firstly, the public dataset GSE143272 was normalized using the “normalize between arrays” function of the limma package. Subsequent analysis of this dataset with R software, unveiled 3399 autophagy related genes comprising 1571 upregulated genes and 1828 downregulated genes, demonstrating differential expression between the epilepsy and control groups in blood, visually displayed in the form of volcano plots (Fig. 4a). A Venn diagram depicted the common DEGs in blood and brain tissue (Fig. 4b). Moreover, violin plots and heatmaps showing the expression of 14 autophagy-related DEGs in peripheral blood samples from individuals with mTLE compared to controls (Fig. 4c and d). Figure 4e presented the correlation of these 14 DEGs of autophagy within the GSE143272 dataset.

### Potential predictive marker of epilepsy in LASSO model

**Fig. 5:**
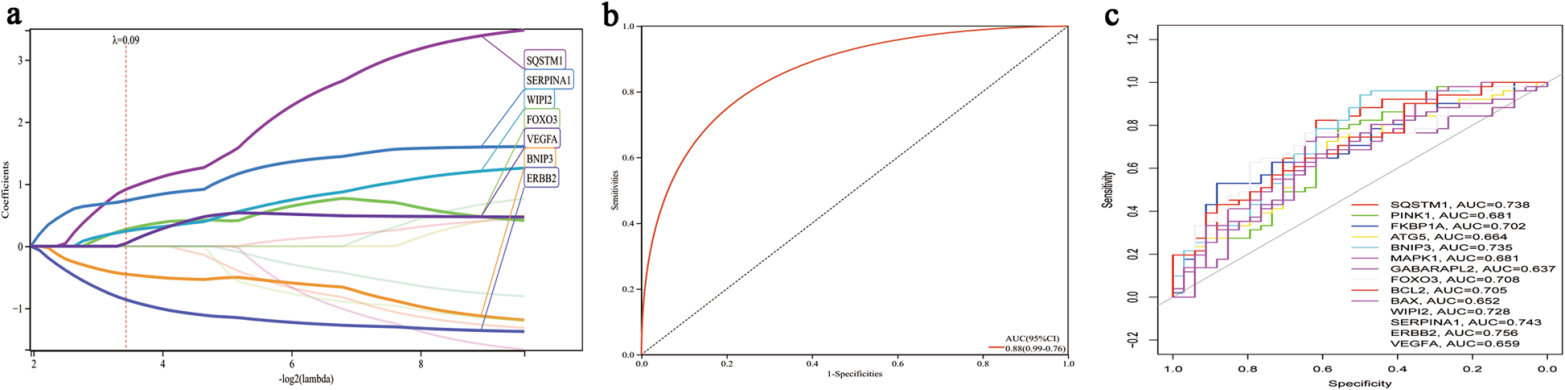
The model for predicting epilepsy. **a** LASSO regression model. **b** ROC curves analysis of dataset. **c** ROC curves analysis of seven genes

As the lambda increases, the coefficients of less important variables tend to shrink to zero, while the coefficients of more important variables are more likely to be retained. Seven genes, namely *SQSTM1*, *BNIP3*, *FOXO3*, *WIPI2*, *SERPINA1*, *ERBB2*, and *VEGFA*, were identified with non-zero regression coefficients, and the value of lambda.min = 0.0925551197393762 was chosen. The gene-based model index was established using the following formula: Index = 0.913938338631802*SQSTM1–0.451510329994845*BNIP3 + 0.271284377754037*FOXO3 + 0.241742532558963*WIPI2 + 0.730859248215233*SERPINA1–0.861673960375642*ERBB2 + 0.0451370867086589*VEGFA (Fig. 5; Table 2)

**Table 2:** The expression of seven genes in blood and brain tissue

Genes	Blood			Brain		
	* P*-value	logFC	expression	* P*-value	logFC	expression
*SERPINA1*	0.00003	0.26540	Up	0.00332	1.23411	Up
*BNIP3*	0.00009	-0.26286	Down	0.00837	-1.09835	Down
*ERBB2*	0.00010	-0.33529	Down	0,00206	1.09161	Up
*SQSTM1*	0.00062	0.13393	Up	0.00028	1.04424	Up
*WIPI2*	0.00078	0.19649	Up	0.00001	-1.05756	Down
*FOXO3*	0.00185	0.19416	Up	0.01642	-1.10002	Down
*VEGFA*	0.00996	0.16619	Up	0.00000	-1.30077	Down

### Data validation using qPCR

**Fig. 6:**
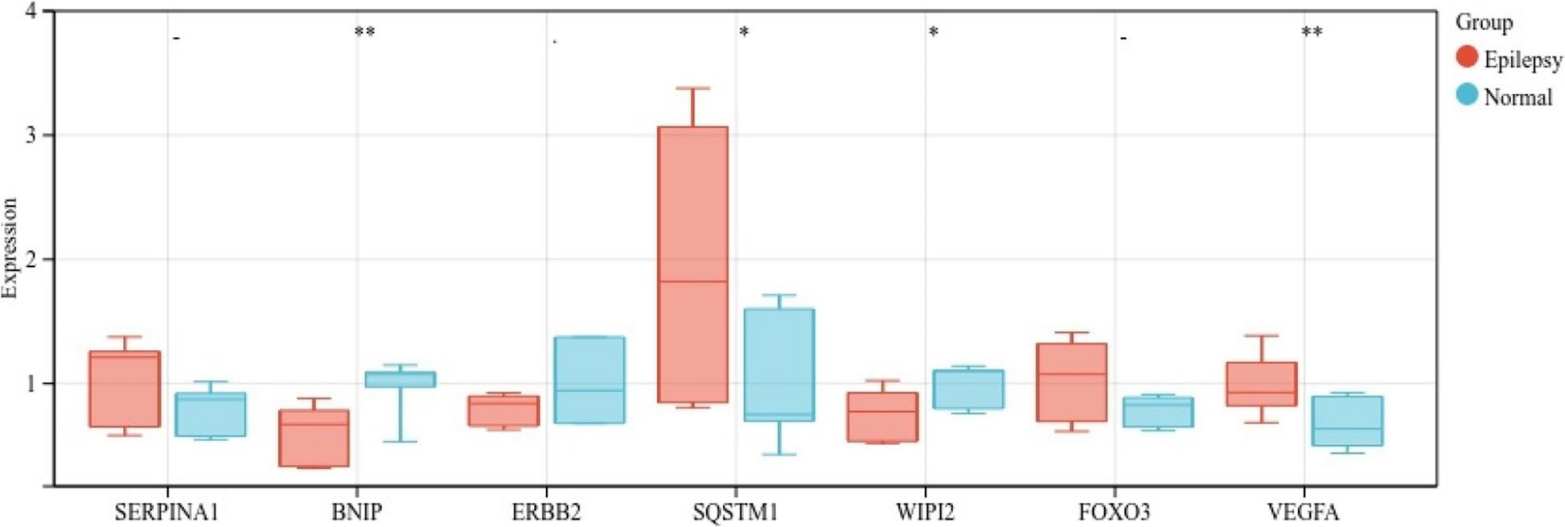
mRNA expression of seven autophagy-related genes

To confirm the reliability of our analysis results, the mRNA expression levels of seven autophagy-related DEGs were further validated by qPCR in samples from our animal model. The results indicated that *SQSTM1* and *VEGFA* exhibited significantly higher expression levels in epilepsy rats compared to the control group. Conversely, the mRNA expression levels of *BNIP* and *WIPI2* were notably decreased. No significant differences was observed in the expression levels of *SERPINA1, ERBB2*, and *FOXO3* (Fig. 6).

## Discussion

Structural, genetic, infectious, metabolic, and immune factors, either singly or in combination, can contribute to epileptogenesis in mTLE [14]. Moreover, the etiologies of mTLE vary among individuals. Accumulating evidence suggests the involvement of autophagy in epileptogenesis related to mTLE. For instance, a previous study demonstrated increased levels of autophagy markers, such as p-mTOR/m-TOR ratio, LC3-II, and phospho-Akt/Akt ratios, along with the occurrence of epileptiform discharge originating from the hippocampus or limbic cortical regions following kainic acid (KA) administration in mice, while establishing temporal lobe epilepsy models [15]. Additionally, another study revealed a sudden increasing in autophagy markers, namely beclin-1 and LC3II/LC3I in pilocarpine epilepsy model [16]. Nevertheless, further comprehensive validation is essential to enhance our understanding of the role of autophagy in the initiation and progression of mTLE.

Recently, a growing number of researches have focused on the relationship between autophagy (including autophagy-related factors or marker proteins) and mTLE. Aronica et al. identified miRNA-146a (miR-146a) as a potential regulator of astrocyte inflammation in TLE, observed in both rats and human subjects [17]. Another study highlighted a significant increase in MAP1LC3, phospho-mTOR/mTOR, and Beclin1 levels following seizure induction in rat models, implying a role of autophagic factors in the pathogenesis of TLE [18]. In addition, emerging evidence has indicated that regulation of Beclin1, a key autophagy-related molecule, could represent a therapeutic approach for managing epilepsy [19]. However, research in this area remains limited and warrants further investigation.

Currently, a multitude of studies have explored autophagy-related genes, such as *WDR45* [9, 20], *CYLD* [21], and *4E-BP2* [22], which have been linked with epilepsy development. Despite this, bioinformatics analysis focusing on autophagy genes in mTLE has been scarce untill now. This study has identified 40 candidate autophagy-related genes in mTLE by bioinformatics analysis, with *ATG16L1* being among the identified genes. A study reported that increasing *ATG16L1* levels through antagomir-223 treatment alleviated epilepsy in KA-treated mice, suggesting the microRNA-223/ATG16L1 pathway may offer a novel treatment option for TLE [23]. Additionally, Castaneda-Cabral et al. found an elevation of *VEGF-A* in microvasculature of patients with TLE, which indicating a connection to the blood-brain barrier dysfunction [24].

Our KEGG analysis has revealed that the identified target genes are predominantly enriched in autophagy, apoptosis, mTOR signaling pathway, and FoxO signaling pathway. In mTLE with HS, there is a loss of neuronal populations and gliosis in the hippocampal region, whereas the temporal cortex exhibits fewer alterations, whose pathogenesis and progression are closely related to autophagy. Gao et al. demonstrated that electroacupuncture treatment for TLE can promote autophagy via the AKT/mTOR signaling pathway [25]. Wen et al. showed that miR-421, which targets MYD88, can suppress autophagy in hippocampal neurons of epileptic mice by down-regulating the TLR/MYD88 pathway [26]. In summary, autophagy plays a key role in neurological disorders, especially epilepsy. Thus, it is essential to reveal the potential biological functions of these DEGs related to autophagy.

The qRT-PCR validation of the expression levels of seven genes, including *SERPINA1*, *BNIP*, *ERBB2*, *SQSTM1*, *WIPI2*, *FOXO3*, and *VEGFA*, corroborated the analysis results. We found that *SQSTM1* and *VEGFA* were significantly upregulated, while *BNIP* and *WIPI2* were significantly downregulated in epilepsy model.

Vascular endothelial growth factor A (VEGFA) is a dimeric glycoprotein secreted by neural tube cells during brain development. Serving as a crucial co-factor between the vascular and nervous systems, VEGFA plays a significant role in endothelial cell proliferation angiogenesis, migration, and neural stem cells proliferation, exerting its biological function by binding to VEGFR2 in the nervous system [27]. It was first isolated and purified from cultured bovine pituitary astrocytes by Ferrara et al. in 1989 [28]. Castaneda-Cabral et al. found elevated VEGFA levels in the neocortex of patients with drug-resistant TLE (DR-TLE) [29], underscoring its potential involvement in TLE pathogenesis. In the rat model of TLE, *VEGFA* has been observed to be up-regulated early in neurons and glial cells in the hippocampus after SE [30, 31]. Furthermore, several studies have highlighted the antiepileptic effects of *VEGFA* in pilocarpine-induced SE rats. VEGFA administration has been shown to reduce neuronal apoptosis post-seizure induction, promote survival and, proliferation of neural stem cells, and facilitate nerve repair. These findings suggest a potential neuroprotective role for *VEGFA* following SE, positioning it as a promising neurovascular molecular target [32–34]. However, *VEGFA* also have a dual role in epilepsy, as it has been linked to blood-brain barrier disruption and epileptogenic inflammation, contributing to the development of epilepsy [35, 36].

*WIPI2*, a human homolog of yeast ATG18, containing three WD-repeats and a seven-bladed b-propeller structure with conserved motifs that facilitates its interaction with other proteins, plays a vital role in phagophore formation, the initial step of autophagy [37]. By interacting with ATG16L1, WIPI2b recruits the ATG12–ATG5–ATG16L1 complex to the phagophore, which is required for LC3 lipidation and autophagosome formation [38, 39]. Dooley et al. discovered that WIPI2 mutants fail to bind ATG16L1, leading to the inhibition of LC3 lipidation and the formation of LC3 puncta [40]. In a case report involving three siblings, it was noted that individuals with homozygous missense *WIPI2* variants exhibited early central nervous system (CNS) involvement, presenting with recurrent and refractory generalized myoclonic and tonic seizures. Furthermore, their EEG results revealed bilateral temporal epileptiform activity [41]. These observations provide evidence linking mutation in *WIPI2*, a major autophagy gene, to the development of epilepsy.

BNIP3, an atypical BH3-only protein share homology with BCL2, plays a dual role in regulating programmed cell death [42] and serving as a potent inducer of autophagy in various cell types [43, 44]. The functional impact of BNIP3-mediated autophagy differs according to the cell type and context, but it is evidently implicated in the pathogenesis of epilepsy. In PTZ-induced epileptic models, the knock-out of *TRPM2* has shown efficacy in ameliorating epilepsy-induced hippocampal pathological damages, probably via the PARP1 downstream signaling pathway involving BNIP3 [45]. Neuronal ceroid lipofuscinoses (NCLs) associated with mutations in the *CLN8* gene can present as progressive epilepsy, with BNIP3 identified as potential protein partners of CLN8, further underlining its role in epileptogenesis [46].

SQSTM1 (p62) is a scaffold protein with PB1 and UBA domains, as well as a TRAF6 binding sequence [47]. In 1998, Shin noticed the unique cytoplasmic punctate structure formed by p62 and created the name sequestosome1 (SQSTM1) based on this characteristic [48]. SQSTM1 is implicated in cell signaling, differentiation, and particularly in clearance of toxic protein aggregates [47]. Depletion of p62 in conjunction with autophagy inhibition has been shown to prevent the accumulation of ubiquitin-positive protein aggregates, suggesting the essential role of p62 in basal autophagy [49]. The significance of p62 as a selective autophagy adaptor for the aggregation and clearance of misfolded proteins is reflected in the epileptogenesis. He et al. found that p62 expression was remarkably high in patients with TLE and in the epileptic hippocampus of mice [23]. In a mouse model of myoclonic epilepsy of Lafora, the absence of p62 impaired glycogen aggregation, exacerbated pathology, and increased susceptibility to epilepsy [50]. These findings provide valuable insights for the development of novel treatment strategies for epilepsy.

In addition, we extracted the mRNA expression profiles of candidate genes to construct the LASSO model, which identified seven genes with non-zero regression coefficients. Among these genes, some have been previously reported to be associated with epilepsy. Analysis of the ROC curve indicated that the LASSO model exhibits a high AUC value, suggesting its potential utility as a biomarker for epilepsy.

In this study, we have unveiled the potential significance of *SQSTM1, VEGFA, BNIP*, and *WIPI2* in autophagy and their subsequent impact on the initiation and progression of mTLE. We propose that these genetic markers could function as diagnostic and therapeutic indicators for clinical application in epileptic patients. Particularly, the impaired degradation of the upregulated *SQSTM1* by autophagy leads to its accumulation [51], contributing to heightened seizure susceptibility [52]. Similarly, *BNIP* has emerged as a reliable indicator of mTLE, with alterations in its gene expression levels causing autophagy dysfunction and consequently elevating the risk of epilepsy [53]. By detecting and analyzing the expression levels of these genes, we anticipate more precise epilepsy risk prediction in patients. This predictive capability could pave the way for the development of targeted and personalized treatment strategies, ultimately improving patient outcomes.

Although several studies have revealed the relationship between autophagy and epilepsy, the precise molecular mechanism remains elusive. Therefore, future research endeavors should delve deeper into elucidating how these genes influence the autophagy process, ultimately leading to the manifestation of epilepsy. This comprehensive exploration should encompass the complex regulation of gene expression, protein-protein interactions, and signal transduction pathways.

This study acknowledges certain limitations. We identified seven candidate autophagy-related genes associated with mTLE through bioinformatics analysis and the mRNA expression levels of seven DEGs were validated in hippocampus from lithium-pilocarpine chronic epilepsy model. However, there was no significant difference in the expression levels of SERPINA1, ERBB2, and FOXO3, which may be attributed to the limited sample size. Furthermore, while the research demonstrated that bioinformatics analysis can reveal critical insights into molecular pathways underlying epilepsy, the candidate key pathways and genes identified through bioinformatics analysis and molecular experiments require further validation. It is essential to determine to what extent downregulation of those genes contributes to the development of epilepsy.

## Conclusions

In conclusion, our study identified 40 potential autophagy-related genes in mTLE were identified through bioinformatics analysis. *SQSTM1*, *VEGFA*, *BNIP*, and *WIPI2* may affect the onset and progression of mTLE by regulating autophagy. These findings may deepen our understanding of mTLE and provide valuable biomarkers for epilepsy.

## Supplementary Information

**Supplementary Materials 1.**

## Data Availability

Authors approved the data and materials availability.

## Abbreviations

BP: Biological process
CC: Cellular component
CNS: Central nervous system
DEGs: Differential expression genes
DR-TLE: Drug-resistant temporal lobe epilepsy
FDR: False detection rate
GO: Gene ontology
HS: Hippocampal sclerosis
KA: Kainic acid
LASSO: Least absolute shrinkage and selection operator
mTLE: Mesial temporal lobe epilepsy
mTOR: Mammalian target of rapamycin
MeSH: Medical subject headings
MF: Molecular function
NCLs: Neuronal ceroid lipofuscinoses
PPIs: Protein-protein interactions
ROC: Receiver operating characteristic
SE: Status epileptics
SQSTM1: Sequestosome1
TLE: Temporal lobe epilepsy
VEGFA: Vascular endothelial growth factor A
