# Supplementary material for: Autophagy-related genes in mesial temporal lobe epilepsy: an integrated bioinformatics analysis
Source: Acta Epileptol. 2024 Apr 25;6:16. doi: 10.1186/s42494-024-00160-9 (PMC11960276; doi:10.1186/s42494-024-00160-9)
Supplement: Supplementary file 1 — Supplementary Materials 1. [file 42494_2024_160_MOESM1_ESM.docx]

**Supplement table 1 The information of genes associated with autophagy in temporal lobe epilepsy.**

| Gene Symbol | *P*-value (Disease) | Fold-Change (MTLE vs. Control) |
| --- | --- | --- |
| *ATG16L1* | -1.08878 | 6.21196×10^-06 |
| *ATG5* | -1.11036 | 2.39435×10^-05 |
| *BAX* | 1.09052 | 1.06644×10^-04 |
| *BCL2* | 1.05906 | 9.84825×10^-03 |
| *BNIP3* | -1.09835 | 8.36758×10^-03 |
| *CANX* | 1.0557 | 1.22019×10^-04 |
| *CAPN1* | -1.05663 | 2.84936×10^-03 |
| *CCL2* | 1.72639 | 1.46833×10^-06 |
| *CD46* | 1.09436 | 1.89524×10^-03 |
| *CFLAR* | 1.26337 | 2.28258×10^-10 |
| *CXCR4* | 1.17246 | 3.52563×10^-05 |
| *ERBB2* | 1.09161 | 2.05765×10^-03 |
| *FKBP1A* | 1.10807 | 4.53746×10^-03 |
| *FOS* | 1.5712 | 5.54899×10^-08 |
| *FOXO1* | 1.11977 | 8.40203×10^-05 |
| *FOXO3* | -1.10002 | 1.64232×10^-02 |
| *GABARAP* | 1.06225 | 3.35297×10^-05 |
| *GABARAPL2* | 1.05735 | 3.37876×10^-03 |
| *GRID2* | 1.16064 | 1.55893×10^-07 |
| *HSPB8* | 1.17814 | 3.11623×10^-05 |
| *IKBKB* | 1.051 | 8.63123×10^-03 |
| *ITPR1* | -1.29419 | 1.58572×10^-08 |
| *LAMP1* | 1.11768 | 5.32205×10^-06 |
| *MAPK1* | 1.08942 | 1.68245×10^-04 |
| *MAPK8* | -1.06102 | 1.26975×10^-02 |
| *MLST8* | -1.06266 | 1.0797×10^-04 |
| *NRG1* | -1.21277 | 1.31643×10^-08 |
| *PIK3C3* | -1.05045 | 1.02276×10^-02 |
| *PINK1* | -1.10129 | 1.61845×10^-07 |
| *RB1CC1* | -1.14736 | 7.77716×10^-08 |
| *SERPINA1* | 1.23411 | 3.32173×10^-03 |
| *SESN2* | -1.11555 | 1.08851×10^-04 |
| *SIRT1* | 1.05111 | 1.1074×10^-02 |
| *SPNS1* | 1.05566 | 1.07303×10^-03 |
| *SQSTM1* | 1.04424 | 2.79341×10^-04 |
| *STK11* | -1.14707 | 4.8213×10^-09 |
| *TP53* | 1.1104 | 1.43885×10^-03 |
| *ULK1* | -1.09834 | 4.67217×10^-06 |
| *VEGFA* | -1.30077 | 1.51403×10^-09 |
| *WIPI2* | -1.05756 | 7.11281×10^-06 |
